# YouTube as a source of patient information on erythema nodosum: an analysis of quality and reliability

**DOI:** 10.1007/s10067-026-08178-9

**Published:** 2026-05-25

**Authors:** Mustafa Gur, Canan Gur, Ahmet Karatas

**Affiliations:** 1 Department of Physical Therapy and Rehabilitation, Division of Rheumatology, Faculty of Medicine, Fırat University, Elazığ, Turkey; 2 Department of Physical Therapy and Rehabilitation, Fethi Sekin City Hospital, Elazığ, Turkey; 3 Department of Internal Medicine, Division of Rheumatology, Faculty of Medicine, Fırat University, Elazığ, Turkey

**Keywords:** Erythema nodosum, Information science, Internet, Social media

## Abstract

**Background:**

YouTube is increasingly being used for health information, however, the quality of videos on erythema nodosum (EN), the most common form of septal panniculitis, remains unclear.

**Objective:**

This study evaluated English-language YouTube videos on EN for their quality and reliability.

**Methods:**

In this cross-sectional study, the search was conducted on November 15, 2025, using the keywords “erythema nodosum,” “erythema nodosum causes,” “erythema nodosum symptoms,” and “erythema nodosum treatment.” The first 100 videos were screened for each search term. After applying the exclusion criteria, 61 videos were included in the analysis and categorized according to uploader type and presentation format. Quality and reliability were measured using the Global Quality Scale, modified DISCERN tool, JAMA Benchmark Criteria, and Patient Education Materials Assessment Tool for Audiovisual Content. The statistical analyses included inter-rater agreement, group comparisons, and correlations.

**Results:**

Among the 61 videos, 47.5% were of low quality, 24.6% were moderate quality, and 27.9% were of high quality. Physician-uploaded videos were generally of higher quality, whereas patient-generated content lacked educational value. Traditional narration and slides dominated, with limited use of animations or patient stories. Viewer engagement, including likes and comments, correlated with quality, but view count did not. The longer and more recent videos tended to score better. The assessment tools showed complementary correlations.

**Conclusion:**

The quality and reliability of YouTube videos on EN are highly variable, with nearly half containing low-quality information. Physician-produced videos were generally more reliable, whereas patient-generated content showed limited educational value. These findings highlight the need for greater expert involvement, improved source transparency, and more engaging evidence-based educational content to reduce misinformation and support patient education on EN.

**Table:** 

**Key Points** • * The quality of English-language YouTube videos on erythema nodosum demonstrates substantial variability, with nearly half categorized as low quality*. • * The highest quality content is mostly produced by physicians, while patient experience videos are educationally insufficient*. • * The numbers of likes and comments show a positive correlation with content quality, whereas view counts are not a reliable indicator of quality*.

## Introduction

With the widespread adoption of information and communication technologies, using the internet to search for health information has become common [1]. The internet is one of the most important sources of health information, followed by health professionals, family members, and mass media. In the future, as more elderly people use the internet and more providers offer online health information, the internet will become even more significant [2]. As the use of digital devices increases and internet access expands, individuals are turning to online platforms for medical issues [3].

Recently, an increasing number of individuals have obtained information through YouTube. Compared to the text format, video is easy, user-friendly, and more efficient for finding certain types of information. These advantages facilitate the process of presenting complex medical information, and YouTube stands out as a health information platform [4]. However, most YouTube content does not undergo a peer-review process. Since the quality and reliability of health content on YouTube can vary significantly, it needs to be evaluated in detail. Understanding the advantages and disadvantages of YouTube as a medical information source is crucial for its effective use [5].

Erythema nodosum (EN) is the most common form of septal panniculitis, characterized by painful, erythematous nodules usually located on the lower extremities. EN can be idiopathic or it may be associated with systemic diseases, infections, medications, pregnancy, and more rarely, malignancies [6, 7]. In rheumatology practice, EN is frequently encountered especially in cases of Behçet’s disease, sarcoidosis, infections, and inflammatory bowel disease [6, 8]. Because EN may be an indicator of an underlying systemic disease and may follow a recurrent course, patients frequently consult online health information. However, incorrect or misleading online information can lead to unnecessary anxiety, delays in medical assessment, or inappropriate self-management attempts.

In recent years, the quality of YouTube content related to various rheumatologic diseases has been increasingly investigated [9–11]. However, to the best of our knowledge, no previous study has evaluated the scientific quality and reliability of YouTube content related to EN. This study evaluated videos related to EN on YouTube. The aim of this study was to identify resources for accessing high-quality videos by analyzing their characteristics. In addition, by comparing video parameters across quality categories, we aimed to obtain enlightening findings.

## Methods

This study was descriptive, cross-sectional analysis aimed at evaluating the quality and reliability of videos related to EN published on YouTube. Because YouTube prioritizes offering personalized results to users, all cookies and browsing histories were deleted. The search was conducted without logging into any user account and in the incognito mode. Thus, biases stemming from personal history or algorithmic suggestions were minimized. The listing was created using the default “relevance-based ranking” setting, which reflects the typical actions of a platform user [12, 13].

All searches were conducted on a single day, November 15, 2025, between 10:00 AM and 2:00 PM (GMT + 3, Turkey), to minimize temporal algorithmic variability. A standard desktop computer with a Turkey-based IP address was used. The browser language was set to English, and no additional filters (e.g., upload date and duration) were applied beyond the default relevance-based ranking. The keywords used were “erythema nodosum,” “erythema nodosum causes,” “erythema nodosum symptoms,” and “erythema nodosum treatment” as query terms. For each keyword, the first 100 videos were screened for inclusion in this study. A typical internet user accesses only a limited portion of a list. This fact is also supported by previous studies [3]. Videos in languages other than English, duplicate videos, irrelevant videos, videos shorter than 1 min or longer than 60 min, and videos with image and sound defects were excluded from the study. Videos shorter than 1 min were excluded due to their limited capacity to provide informative content and their inability to be evaluated by video quality scales because of insufficient medical information. Videos longer than 60 min were generally excluded because they usually consisted of conference recordings or professional training materials, differing from the typical YouTube content aimed at public education. During the data cleaning process, duplicate videos were removed to ensure dataset accuracy. Initially, duplicates were identified by comparing YouTube video IDs across different keyword searches. Additionally, since the same content may be re-uploaded by different users under different video IDs, videos were further screened for content similarity based on title, duration, and visual inspection. When substantially identical content was identified, only one version was retained in the final dataset.

For the video evaluation process, the two researchers initially scored the videos independently. Inconsistencies were identified by comparing independent ratings. For videos with differing evaluations, the two primary raters first discussed the case to attempt consensus. If disagreement persisted, a third senior researcher independently reviewed the video and assigned a final score, which was used for analysis. The agreement between the two primary raters’ initial scores for the Global Quality Scale (GQS) was assessed using Cohen’s kappa coefficient.

### Video features

Through YouTube, data were recorded on the number of views, likes, and comments, duration (in seconds), the time elapsed between upload date and analysis date, as well as daily view, like, and comment metrics for the videos. Daily metrics were calculated by dividing the total count of views, likes, or comments by the number of days elapsed since the video upload date to the date of analysis (November 15, 2025). The formula used was: daily metric = total count/number of days since upload. Based on the formatting method, videos were grouped as those with the narrator only, those presenting patient experiences, those including animation, and slide presentations.

### Video sources

The videos were divided into three groups according to the self-declared identity of the uploader:

- *Physicians*: Uploaders who clearly state in the video content or channel description that they are medical doctors
- *Patients*: Individuals sharing their personal experiences with EN without indicating any professional healthcare identity
- *Unspecified sources*: Videos in which the uploader does not explicitly identify themselves as a healthcare professional or patient. If there was no clear statement about professional identity in the channel description, external links, or video content, the video was classified into this category.

### Content assessment

The GQS, a widely recognized measurement tool for evaluating the educational quality of online resources, was used to determine the overall quality of videos. The GQS rates content on a 5-point grading system, where 1 point indicates the lowest quality and 5 points the highest quality [14, 15].

- Low quality (score 1–2): Videos at this level contain significant errors, inconsistencies, or major information gaps that reduce their usability for instructional purposes.
- Moderate quality (score 3): While these videos possess a certain degree of educational value, they may lack depth, clarity, or comprehensive coverage.
- High quality (score 4–5): Videos in this category have accurate content, meticulous organization, and instructional value; therefore, they are considered to be effective and reliable educational material.

A modified DISCERN tool was used to assess the reliability of the videos. This tool comprehensively examines various criteria such as clarity, understandability, impartiality, objectivity, and the inclusion of references and supplemental resources. This assessment method is based on binary (yes/no) questions, awarding 1 point for each affirmative answer and 0 points for each negative answer. The maximum possible total in this system was 5 [16]. The five items of the modified DISCERN tool are as follows: (1) Is the video clear, concise, and understandable? (2) Are valid sources cited? (Is the information based on a specific reference?) (3) Is the information provided balanced and unbiased? (4) Are additional sources of information listed for patient reference? (5) Does the video address areas of uncertainty or controversy?

The JAMA Benchmark Criteria is a scale used to evaluate the accuracy and quality of online health information. These criteria systematically examine essential elements that determine the credibility of internet-based information sources, such as authorship, attribution, disclosure, and currency. Each fulfilled criterion was awarded 1 point, while non-fulfilled criteria received 0 points. Consequently, the total score for each video ranged between 0 and 4, with a score of 4 indicating the highest quality and reliability [17].

The Patient Education Materials Assessment Tool for Audiovisual Content (PEMAT-A/V) provides a structured approach to assessing the effectiveness of medical educational materials focused on audio and visual content. This tool addresses two main dimensions: understandability and actionability. Understandability evaluates how clearly, systematically, and appropriately information is presented making it easy for the target audience to follow and interpret through the use of visuals. Actionability examines whether the material offers clear, actionable, and directive recommendations that can help individuals manage their health conditions. Evaluation results are reported as percentage values indicating which standards the material meets in terms of overall accessibility and practical usability [18].

### Ethics approval

This study is based solely on the analysis of publicly available YouTube content using a document analysis method. No direct interaction with participants was conducted, and no intervention or personal data collection took place during the research process. All analyzed materials consist of content that users have made publicly accessible. Therefore, the study does not involve human subjects in an experimental or observational manner and does not require approval from an ethics committee.

### Statistical analyses

Statistical analyses were conducted using version 29.0 of the SPSS software (SPSS Inc., Chicago, IL, USA). Before beginning the analyses, the Shapiro–Wilk normality test was applied to assess the distribution characteristics of the data set. Descriptive statistics were given as median, frequency (*n*), and percentage (%) values. The Kruskal–Wallis test was used to compare the three predetermined groups. The Spearman rho correlation analysis was performed to assess relationships between variables. The Kappa coefficient was calculated to determine inter-rater reliability. In all analyses, *p* < 0.05 was considered the threshold for statistical significance.

## Results

**Fig. 1:**
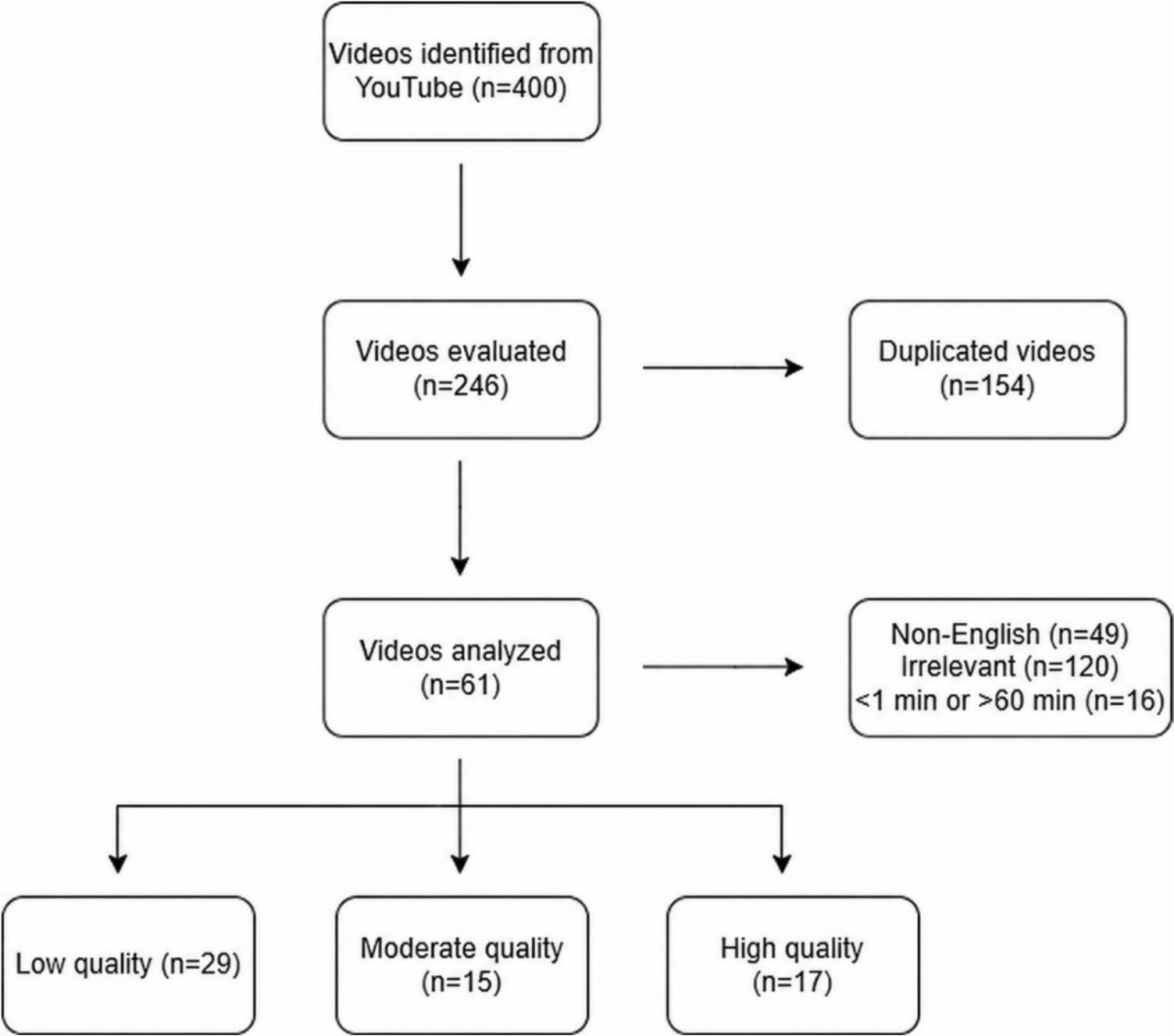
Visualization of the inclusion and exclusion process of videos

A total of 400 videos were identified by evaluating the first 100 videos listed under YouTube’s default relevance-based ranking for each search term. Three hundred thirty-nine videos that did not meet the inclusion criteria were excluded from the study, and 61 videos were included for evaluation. Detailed information on the sample selection process is presented in Fig. 1. The median duration of the videos was 207 (66–1848) s. The median number of views, likes, and comments were 2530 (27–95,025), 18 (0–1221), and 2 (0–167), respectively. While 37.7% (*n* = 23) of the videos were presented solely with a narrator, 11.5% (*n* = 7) featured patient experiences, 11.5% (*n* = 7) included animations and drawings, and 39.3% (*n* = 24) consisted of slide presentations. The main characteristics of the videos are summarized in Table 1.

**Table 1:** Characteristics of the videos included in the analysis

Video features	Median (range)
Video length (seconds)*	207 (66–1848)
Total view count*	2530 (27–95,025)
Total likes*	18 (0–1221)
Total comments*	2 (0–167)
Time since upload (days)*	1466 (134–5752)
Average daily views*	1.46 (0.04–123.4)
Average daily likes*	0.01 (0–1.81)
Average daily comments*	0.002 (0–0.20)
**Format of presentation (*n*; %)**	
Video containing only narrator (s)	23 (37.7)
Video containing patient experiences	7 (11.5)
Animation	7 (11.5)
Narrating with a slide presentation	24 (39.3)
**Uploader category (*n*; %)**	
Physician	27 (44.3)
Patient	7 (11.4)
Unspecified source	27 (44.3)

^*^Data are expressed as median (minimum–maximum)

According to GQS rankings, videos were categorized into three groups: low, moderate, and high quality. Of the total videos, 47.5% (*n* = 29) were evaluated as low quality, 24.6% (*n* = 15) as moderate quality, and 27.9% (*n* = 17) as high quality. The Cohen’s kappa score for inter-rater agreement on GQS-based quality categorization was 0.72, which corresponds to substantial agreement. Of the 61 videos evaluated, the two primary raters reached consensus on 53 (86.9%), while discordant ratings were observed in 8 videos (13.1%). In all discordant cases, adjudication by the third senior reviewer was required to reach a final score.

Video sources were also classified by quality. While 40.7% of the videos uploaded by physicians were of high quality, only 22.2% of those uploaded by independent users were of high quality. None of the videos uploaded by patients was of high quality. The evaluation of video quality according to video sources is summarized in Table 2.

**Table 2:** Categorization of videos according to source and presentation format, *N* (%)

Source	Low quality	Moderate quality	High quality	Total
Physician	7 (25.9)	9 (33.3)	11 (40.7)	27
Patient	7 (100)	0 (0)	0 (0)	7
Unspecified source	15 (55.6)	6 (22.2)	6 (22.2)	27
**Presentation format**				
Video containing only narrator (s)	12	5	6	23
Video containing patient experiences	7 (100)	0 (0)	0 (0)	7
Animation	2 (28.6)	3 (42.8)	2 (28.6)	7
Narrating with a slide presentation	8 (33.3)	7 (29.2)	9 (37.5)	24

When quality groups were compared based on daily likes and comments, significant differences were observed (*p* < 0.05). Low-quality videos had fewer likes and comments (Table 3).

**Table 3:** Comparison of the video parameters between the low-quality, moderate, and high-quality groups

Variable	Low quality	Moderate quality	High quality	*p*
Average daily views	1.06 (0.04–8.01)	2.02 (0.19–30.79)	1.57 (0.05–123.41)	0.17
Average daily likes	0.01 (0–0.10)	0.01 (0–0.99)	0.05 (0–1.81)	0.02*
Average daily comments	0 (0–0.04)	0 (0–0.09)	0 (0–0.2)	0.03*

Values are presented as median (minimum–maximum). *p < 0.05

Correlation analyses were conducted between GQS and the other video evaluation tools, and statistically significant correlations were found (*p* value for JAMA Benchmark Criteria 0.02, *p* < 0.001 for other values, and rho = 0.607 for modified DISCERN, rho = 0.307 for JAMA Benchmark Criteria, rho = 0.840 for PEMAT-A/V Understandability, and rho = 0.672 for PEMAT-A/V Actionability). In addition, correlations between video parameters and scores from video evaluation tools were analyzed. The results showed positive correlations between video duration and GQS and PEMAT-A/V Understandability scores; between the number of days and PEMAT-A/V Understandability scores; and between the daily number of likes and PEMAT-A/V Understandability scores (*p* < 0.01). The data on daily number of views and daily number of likes are summarized in Table 4.

**Table 4:** Correlation analysis between content scores and video parameters

	GQS	Modified DISCERN questionnaire	JAMA Benchmark criteria	PEMAT-A/V understandability	PEMAT-A/V actionability
Video length	0.464**	0.265*	0.296*	0.344**	0.244
Time since upload (days)	− 0.279*	− 0.290*	− 0.098	− 0.386**	− 0.232
Average daily views	0.240	− 0.017	0.023	0.189	0.095
Average daily likes	0.326*	0.142	0.212	0.333**	0.144
Average daily comments	0.267*	0.030	0.194	0.225	0.194

GQS, Global Quality Scale; JAMA, *Journal of the American Medical Association*; PEMAT-A/V, Patient Education Materials Assessment Tool for Audio/Visual Materials

^**^p < 0.01; ^*^p < 0.05

## Discussion

The evaluation of videos on YouTube related to EN provides significant insights into the overall quality level and structural characteristics of health-related content on this widely used platform. Our findings reveal a marked variability in the informational quality of online videos about EN, with approximately half containing low-quality or incomplete information. Notably, videos uploaded by physicians had higher quality scores, whereas those uploaded by patients were associated with lower informational quality.

The fact that most videos were presented either solely with a narrator or as slide-based presentations suggests that content creators predominantly adopt traditional, one-way information delivery methods. Although such presentations can be advantageous in terms of information density, they may be limited in sustaining viewer attention and explaining complex clinical processes through visualization. In contrast, videos featuring patient experiences offer a unique contribution by illustrating the impact of the disease on daily life, creating emotional connections with viewers, and fostering empathy. The relatively small number of animation- and drawing-based contents is also noteworthy, as these visual tools can enable more comprehensible and memorable transmission of complex pathophysiological and clinical concepts. Indeed, systematic reviews report that visual-based educational approaches significantly improve the perception and understanding of health information compared to solely written or verbal information delivery [19].

Studies in the literature evaluating the quality and reliability of YouTube content on rheumatologic diseases have shown substantial heterogeneity in the presentation of information in online videos. Studies examining YouTube videos on gout, familial Mediterranean fever, spondyloarthritis, pregnancy in rheumatoid arthritis, and exercise in rheumatoid arthritis have reported relatively higher rates of moderate and high-quality content [10, 15, 20–22]. In contrast, research evaluating videos on inflammatory back pain and autoinflammatory diseases has shown that most of the content was of low quality [23, 24]. This difference may be related to the higher prevalence of certain rheumatologic diseases in the community, greater patient awareness, and increased participation of healthcare professionals and academic institutions in digital content production in these areas. However, in rarer or diagnostically complex diseases, the limited production of information may result in content being mainly experience-based or relying on unverified sources. This suggests that factors such as disease prevalence, level of clinical complexity, and expert visibility may affect the quality of online health information. These results support the notion that YouTube content regarding rheumatologic diseases lacks consistent standardization in terms of quality and that the informational quality can vary significantly depending on the subject and the qualifications of the content creator.

The “unspecified source” category, which constitutes 44.3% of the videos included in the study, requires a more detailed evaluation. This heterogeneous group may include health information websites, channels belonging to commercial or pharmaceutical companies, artificial intelligence-generated content, and other unverified sources whose levels of reliability may vary. The lack of explicit source information in these videos may partially explain the variability observed in quality scores and highlights the difficulty users may experience in assessing the reliability of online health information. Considering the increasing prevalence of anonymously produced or algorithm-generated medical content, the high proportion of videos in this category underscores the importance of source transparency and expert verification on digital health platforms.

When daily interaction parameters were compared between quality groups of videos, statistically significant differences were found in the numbers of likes and comments, suggesting a possible relationship between viewer engagement and content quality. In particular, the lower daily like and comment counts for low-quality videos may indicate that users are more likely to interact with higher-quality content. However, the absence of a significant difference in daily viewing numbers suggests that watching behavior may be influenced more by platform dynamics such as title, visual appeal, or algorithmic recommendations rather than content quality. These findings highlight the potential usefulness of user engagement metrics as indirect indicators of video quality [3].

The significant correlations found between GQS and other video assessment tools such as Modified DISCERN, JAMA criteria, and PEMAT-A/V indicate that these instruments offer consistent and mutually reinforcing frameworks for evaluating health-related content on YouTube. These relationships suggest that different assessment frameworks overlap to some extent in measuring fundamental quality dimensions such as informational accuracy, reliability, transparency, and comprehensibility. However, since each tool focuses on different components of content quality, these instruments may be considered methodologically complementary. Therefore, using these scales together has the potential to more comprehensively reveal the multidimensional quality structure of online health videos compared to assessments based on a single tool.

Positive and statistically significant correlations were found between video duration and quality metrics (especially GQS, JAMA, and PEMAT-A/V Understandability). This finding may suggest that longer videos allow for more comprehensive coverage of pathophysiology, differential diagnosis, and management, rather than being inherently superior in quality. It is also noteworthy that longer videos may more closely resemble structured educational presentations rather than informal content, which could influence scoring on assessment tools designed to evaluate educational quality. In contrast, negative correlations were found between upload time and certain quality scores. The tendency for recently uploaded videos to receive higher GQS, Modified DISCERN, and PEMAT-A/V Understandability scores could indicate improvements in content production standards over time or that more recent content is prepared with greater quality awareness. The lack of a significant correlation between the number of daily views and quality scores supports the idea that view counts are not a reliable indicator of content quality. On the other hand, the positive correlation of daily likes with GQS and PEMAT-A/V Understandability, and of daily comments with GQS, suggests that users tend to engage more with videos that are more understandable and of higher overall quality. However, considering that these engagement metrics are affected by numerous platform dynamics, it should not be forgotten that these relationships must be evaluated observationally rather than causally.

This study has some limitations. Firstly, the analysis was conducted using only certain keywords and included only English-language videos; this may have excluded content accessible via different search terms or videos in other languages from evaluation. The exclusion of videos longer than 60 min may have led to the omission of in-depth educational content such as conference recordings or comprehensive lectures, which could potentially offer high-quality and detailed information on EN. Although such content differs from typical patient-oriented YouTube videos, their exclusion may have introduced selection bias toward shorter, less detailed formats. Additionally, because YouTube’s search and recommendation algorithms can vary according to user behavior and time, even though cookies were cleared and incognito mode was used to reduce personalization effects, the results cannot be guaranteed to fully reflect the experience of all users. Despite these precautions, the dynamic and personalized nature of YouTube’s relevance-based ranking system inherently limits the reproducibility of the search results. Since the study has a cross-sectional design, findings reflect only a specific time frame of the platform’s dynamic nature, as video view counts, likes, comments, and even content may change over time. Considering that the searches were conducted using an IP address based in Turkey and targeting only English-language videos, it should be taken into account that the geographic mismatch may have affected the ranking and visibility of the included videos due to the YouTube algorithm prioritizing content based on regional relevance and user location and that content more prominently featured in English-speaking countries may not have been adequately represented in the study. The influence of health literacy levels on content interpretation was not assessed, which may affect how different audiences perceive and benefit from the evaluated videos. The uploader classification was based on self-declared identity, which carries an inherent risk of misclassification; some uploaders may not accurately disclose their professional credentials, and the absence of a verification mechanism may have led to potential misattribution of video sources.

In the future, studies that include videos in different languages and cover searches conducted from various geographic regions may increase the generalizability of the findings. Additionally, repeated analyses to be conducted at different time points using standardized search strategies may help mitigate the impact of YouTube’s dynamic recommendation algorithms and strengthen the validity and reproducibility of the results.

The evaluation of YouTube videos related to EN has revealed that the available content exhibits marked differences in terms of quality, reliability, and educational value. While some videos provide useful information, a significant portion of the content contains insufficient or low-quality information. This highlights the existing challenges in accessing accurate health information on open-access digital platforms. Our findings emphasize the importance of increasing expert participation in online health communication and developing more accessible, evidence-based educational materials. In the future, educational strategies that incorporate animations, visual teaching tools, and patient-centered explanations, as well as collaborations among health professionals, academic institutions, and patient organizations, may enhance the quality and reliability of online information on EN.

## Data Availability

The datasets generated and/or analyzed during the current study are available from the corresponding author on reasonable request.

## References

[1] Jia X, Pang Y, Liu LS (2021) Online health information seeking behavior: a systematic review. Healthcare (Basel). 10.3390/healthcare9121740 34946466 10.3390/healthcare9121740 PMC8701665

[2] Wollmann K, der Keylen PV, Tomandl J, Meerpohl JJ, Sofroniou M, Maun A, Voigt-Radloff S (2021) The information needs of internet users and their requirements for online health information-a scoping review of qualitative and quantitative studies. Patient Educ Couns 104(8):1904–1932. 10.1016/j.pec.2021.01.020 33563502 10.1016/j.pec.2021.01.020

[3] Assylbek MI, Zimba O, Akyol A, Yessirkepov M, Kocyigit BF (2025) YouTube as a source of information for stroke rehabilitation: a cross-sectional analysis of quality and reliability of videos. Rheumatol Int 45(4):77. 10.1007/s00296-025-05832-4 40119932 10.1007/s00296-025-05832-4 PMC11929695

[4] Oh SW (2023) YouTube, health information, and health literacy. Korean J Fam Med 44(6):301–302. 10.4082/kjfm.44.6E 37989277 10.4082/kjfm.44.6E PMC10667071

[5] Zimba O, Gasparyan AY, Qumar AB (2024) Ethics for disseminating health-related information on YouTube. J Korean Med Sci 39(7):e93. 10.3346/jkms.2024.39.e93 38412615 10.3346/jkms.2024.39.e93 PMC10896703

[6] Kisacik B, Onat AM, Pehlivan Y (2013) Multiclinical experiences in erythema nodosum: rheumatology clinics versus dermatology and infection diseases clinics. Rheumatol Int 33(2):315–318. 10.1007/s00296-012-2413-5 22441968 10.1007/s00296-012-2413-5

[7] Perez-Garza DM, Chavez-Alvarez S, Ocampo-Candiani J, Gomez-Flores M (2021) Erythema nodosum: a practical approach and diagnostic algorithm. Am J Clin Dermatol 22(3):367–378. 10.1007/s40257-021-00592-w 33683567 10.1007/s40257-021-00592-w PMC7938036

[8] Garcia-Porrua C, Gonzalez-Gay MA, Vazquez-Caruncho M, Lopez-Lazaro L, Lueiro M, Fernandez ML, Alvarez-Ferreira J, Pujol RM (2000) Erythema nodosum: etiologic and predictive factors in a defined population. Arthritis Rheum 43(3):584–592. 10.1002/1529-0131(200003)43:3<584::AID-ANR15>3.0.CO;2-6 10728752 10.1002/1529-0131(200003)43:3<584::AID-ANR15>3.0.CO;2-6

[9] Singh AG, Singh S, Singh PP (2012) YouTube for information on rheumatoid arthritis–a wakeup call? J Rheumatol 39(5):899–903. 10.3899/jrheum.111114 22467934 10.3899/jrheum.111114

[10] Elangovan S, Kwan YH, Fong W (2021) The usefulness and validity of English-language videos on YouTube as an educational resource for spondyloarthritis. Clin Rheumatol 40(4):1567–1573. 10.1007/s10067-020-05377-w 32880051 10.1007/s10067-020-05377-w

[11] Karakoyun A, Yildirim A (2021) YouTube videos as a source of information concerning Behcet’s disease: a reliability and quality analysis. Rheumatol Int 41(12):2117–2123. 10.1007/s00296-021-05009-9 34590188 10.1007/s00296-021-05009-9

[12] Sui W, Sui A, Rhodes RE (2022) What to watch: practical considerations and strategies for using YouTube for research. Digit Health 8:20552076221123707. 10.1177/20552076221123707 36105625 10.1177/20552076221123707 PMC9465614

[13] Zhaksylyk A, Yessirkepov M, Akyol A, Kocyigit BF (2024) YouTube as a source of information on public health ethics. J Korean Med Sci 39(7):e61. 10.3346/jkms.2024.39.e61 38412608 10.3346/jkms.2024.39.e61 PMC10896704

[14] Kocyigit BF, Akyol A (2021) YouTube as a source of information on COVID-19 vaccination in rheumatic diseases. Rheumatol Int 41(12):2109–2115. 10.1007/s00296-021-05010-2 34562126 10.1007/s00296-021-05010-2 PMC8475344

[15] Onder ME, Zengin O (2021) YouTube as a source of information on gout: a quality analysis. Rheumatol Int 41(7):1321–1328. 10.1007/s00296-021-04813-7 33646342 10.1007/s00296-021-04813-7 PMC7917371

[16] Korkmaz M, Altin YF, Yagci TF, Korkmaz MD, Akgul T (2024) Is YouTube a reliable and quality source on unilateral biportal endoscopic spine surgery? A cross-sectional study. World Neurosurg 187:e181–e188. 10.1016/j.wneu.2024.04.063 38642831 10.1016/j.wneu.2024.04.063

[17] Eksi Ozsoy H (2021) Evaluation of YouTube videos about smile design using the DISCERN tool and Journal of the American Medical Association benchmarks. J Prosthet Dent 125(1):151–154. 10.1016/j.prosdent.2019.12.016 32085870 10.1016/j.prosdent.2019.12.016

[18] Karatas L, Utkan Karasu A, Demirsoy N (2024) Is YouTube a sufficient and reliable source to inform patients about cardiac rehabilitation?: a cross-sectional study. J Cardiopulm Rehabil Prev 44(4):239–247. 10.1097/HCR.0000000000000864 38875164 10.1097/HCR.0000000000000864

[19] Galmarini E, Marciano L, Schulz PJ (2024) The effectiveness of visual-based interventions on health literacy in health care: a systematic review and meta-analysis. BMC Health Serv Res 24(1):718. 10.1186/s12913-024-11138-1 38862966 10.1186/s12913-024-11138-1 PMC11165863

[20] Yagiz B, Coskun BN, Halil EY, Dalkilic E, Pehlivan Y (2023) The efficacy and reliability of English YouTube videos as a source of information for pregnant rheumatoid arthritis patients. Clin Rheumatol 42(12):3311–3320. 10.1007/s10067-023-06780-9 37814092 10.1007/s10067-023-06780-9

[21] Sari F, Bazancir Apaydin Z, Sari S (2025) Assessment of reliability and quality of YouTube(R) exercise videos in people with rheumatoid arthritis. Physiother Theory Pract 41(2):362–369. 10.1080/09593985.2024.2334753 38536002 10.1080/09593985.2024.2334753

[22] Coskun BN, Yagiz B, Giounous Chalil E, Dalkilic E, Pehlivan Y (2024) The usefulness and reliability of English-language YouTube videos as a source of knowledge for patients with familial Mediterranean fever. PeerJ 12:e16857. 10.7717/peerj.16857 38390386 10.7717/peerj.16857 PMC10883151

[23] Kara M, Ozduran E, Mercan Kara M, Hanci V, Erkin Y (2024) Assessing the quality and reliability of YouTube videos as a source of information on inflammatory back pain. PeerJ 12:e17215. 10.7717/peerj.17215 38618560 10.7717/peerj.17215 PMC11016243

[24] Sasse M, Ohrndorf S, Palmowski A, Wagner AD, Burmester GR, Pankow A, Krusche M (2023) Digital health information on autoinflammatory diseases: a YouTube quality analysis. Rheumatol Int 43(1):163–171. 10.1007/s00296-022-05243-9 36374326 10.1007/s00296-022-05243-9 PMC9839787

